# Analysis of lytic polysaccharide monooxygenase activity in thermophilic fungi by high-performance liquid chromatography–refractive index detector

**DOI:** 10.3389/fmicb.2022.1063025

**Published:** 2022-11-22

**Authors:** Weishuai Yu, Jie Yu, Duochuan Li

**Affiliations:** Department of Mycology, Shandong Agricultural University, Taian, Shandong, China

**Keywords:** thermophilic fungi, auxiliary activity family 9, lytic polysaccharide monooxygenase, enzyme activity assay, high-performance liquid chromatography

## Abstract

**Introduction:**

Most current methods for analysing the activity of LPMO are based on the quantification of H_2_O_2_, a side product of LPMO; however, these methods cannot assay the LPMO activity of thermophilic fungi because of the low thermostability of H_2_O_2_. Therefore, we present a high-performance liquid chromatography–refractive index detector (HPLC-RID) method to assay the LPMO activity of the thermophilic fungus *Thermoascus aurantiacus*.

**Results:**

According to the established method, the specific activities of nTaAA9A C1 and C4 oxidation were successfully analysed and were 0.646 and 0.574 U/mg, respectively. By using these methods, we analyzed the C1 and C4 oxidation activities of the recombinant TaAA9A (rTaAA9A) and mutated rTaAA9A (Y24A, F43A, and Y212A) expressed in Pichia pastoris. The specific activities of rTaAA9A C1 and C4 oxidation were 0.155 and 0.153 U/mg, respectively. The specific activities of Y24A, F43A, and Y212A C1 and C4 oxidation were 0.128 and 0.125 U/mg, 0.194 and 0.192 U/mg, and 0.097 and 0.146 U/mg, respectively.

**Discussion:**

In conclusion, the method can assay the LPMO activity of thermophilic fungi and directly target C1 and C4 oxidation, which provides an effective activity assay method for LPMOs of thermophilic fungi.

## Introduction

Recently, many studies have shown that oxidative enzymes are involved in the degradation of cellulose. It was demonstrated that the glycoside hydrolase 61 (GH61) enhancement of cellulase activity follows an oxidative mechanism (Karkehabadi et al., 2008; Quinlan et al., 2011), similar to a structurally related chitin-binding protein, CBP21 (Vaaje-Kolstad et al., 2010; Aachmann et al., 2012). Structural and functional studies have shown that GH61 enzymes are Cu^2+^-dependent oxidative enzymes (Koseki et al., 2008; Quinlan et al., 2011). GH61 glycoside hydrolases was reclassified Auxiliary Activity 9 (AA9) and are characterized as lytic polysaccharide monooxygenases (LPMOs) containing highly conserved histidine residues as Cu^2+^ ligands (Levasseur et al., 2013). GH61 enzymes can oxidize C1 carbon of pyranose ring, as well as the C4 and C6. C1 oxidation results in the formation of sugar lactones, and C4 and C6 oxidation results in the formation of ketoaldoses (Phillips et al., 2011; Quinlan et al., 2011; Beeson et al., 2012; Horn et al., 2012). However, some studies have shown that C6 oxidation also exists in LPMO, but the current classification standards do not mention the C6 oxidation, which leads to C6-oxidation in the LPMO field is not well established (Chen et al., 2018, 2019). Another important feature of LPMO is that the N-terminal histidine is methylation-modified and that N-terminal histidine methylation is only observed on LPMO expressed in filamentous fungi, where it is essential for copper binding and enzyme activity (Petrović et al., 2018). It also shows that the production of proteins containing this unique modification has great biotechnological potential (Tanveer et al., 2022).

Currently, in the Carbohydrate-Active Enzymes (CAZy) database, LPMOs are classified into eight auxiliary activity (AA) families: families AA9-AA11 and AA13-AA17.^1^ Among them, LPMOs of AA9 family contains only sequences of fungal origin, contains several substrate specificities and targets mainly glucose-based polymers such as cellulose, cello-oligosaccharides and hemicelluloses, which show potential applications in the secondary biorefinery industry (Levasseur et al., 2013; Bissaro et al., 2018; Filiatrault-Chastel et al., 2019; Vandhana et al., 2022). Several LPMOs in the AA9 family have been characterized from different fungi, such as *Neurospora crassa* (Phillips et al., 2011; Beeson et al., 2012; Kittl et al., 2012; Li et al., 2012), *Phanerochaete chrysosporium* (Westereng et al., 2011; Wu et al., 2013), *Podospora anserina* (Bennati-Granier et al., 2015), and *Sporotrichum thermophile* (Dimarogona et al., 2012). However, accurate detection of their activity is difficult due to the insolubility of the substrates, the diversity of the side reactions and the complexity of the products. To address this problem, activity analysis methods based on high precision equipment such as high-performance anion exchange chromatography (HPAEC) have been established. HPAEC is currently the most widely used method for the analysis of C1 oxidation products, as it is stable under alkaline eluent and therefore it can be separated (Wang et al., 2021). However, C4 oxidation products are unstable and can be decomposed during separation, resulting in additional products (Westereng et al., 2016). A high-throughput activity analysis method was established using the firm binding of nickel ions to glyoxalate, the cleavage product of LPMO on glycosidic chains (Wang et al., 2018). This method directly targets LPMO activity but can only detect LPMO with stereoselectivity for C1 oxidation, not for C4 oxidation. Recently, a horseradish peroxidase (HRP) colorimetric method was developed based on gluco-oligosaccharide oxidase (GOOX) to determine AA9 LPMO activity (Wu et al., 2022). However, the use of the HRP colourimetric method can be somewhat limited if the sample is contaminated with endoglucanase. This method is suitable for those derived from recombinant LPMO expressed in *Escherichia coli* or fully purified LPMO. Accurate detection of LPMO activity is mainly detected by HPLC or MS methods, other activity methods are based on the reduction of LPMO by different reducing agents or the formation of H_2_O_2_ from LPMO as a by-product, or the conversion of the released product into a colorimetric substance by other methods. However, since thermophilic fungi generally grow at temperatures higher than 50°C, and H_2_O_2_ is unstable at this temperature, it is difficult to accurately detect LPMO activity in thermophilic fungi. In light of recent findings related to the better suitability of H_2_O_2_ as a co-substrate for LPMO relative to O_2_, the use of HPLC methods was also necessary (Bissaro et al., 2017). Therefore, current methods are unable to accurately assay the LPMO activity of thermophilic fungi.

In recent years, thermophilic fungi have attracted increasing attention because of their ability to secrete enzymes that decompose biomass at high temperatures (McClendon et al., 2012). LPMOs produced by thermophilic fungi have been identified as promising enzymes to increase the rate of conversion of biomass, such as lignocellulose, into biofuels (Berka et al., 2011; McClendon et al., 2012). The screening of LPMOs with superior activity from the AA9 family, which is widely distributed in the fungi, will effectively facilitate their industrial application. An accurate and comprehensive method for the determination of LPMO activity can not only screen out highly active LPMOs but also better study their properties and catalytic mechanisms. However, the analysis of LPMO activity remains difficult due to the complexity of the products, self-oxidation of the reactants, and lack of standard analytical methods (Loose et al., 2014; Breslmayr et al., 2018; Telitsin et al., 2020). Therefore, it is crucial to establish a method to assay the LPMO activity of thermophilic fungi.

Here, we present a new quantitative method to assay LPMO (TaAA9A) activity of the thermophilic fungus *Thermoascus aurantiacus*. This method was used to determine the activity of LPMO by chemically treating the C1 and C4 oxidation products of LPMO and quantifying the resulting gluconic acid and galactose with high-performance liquid chromatography–refractive index detector (HPLC-RID). In this way, the influence of temperature, impurities and other factors can be avoided, and the difference between C1 and C4 oxidation can be analyzed.

**Figure 1:**
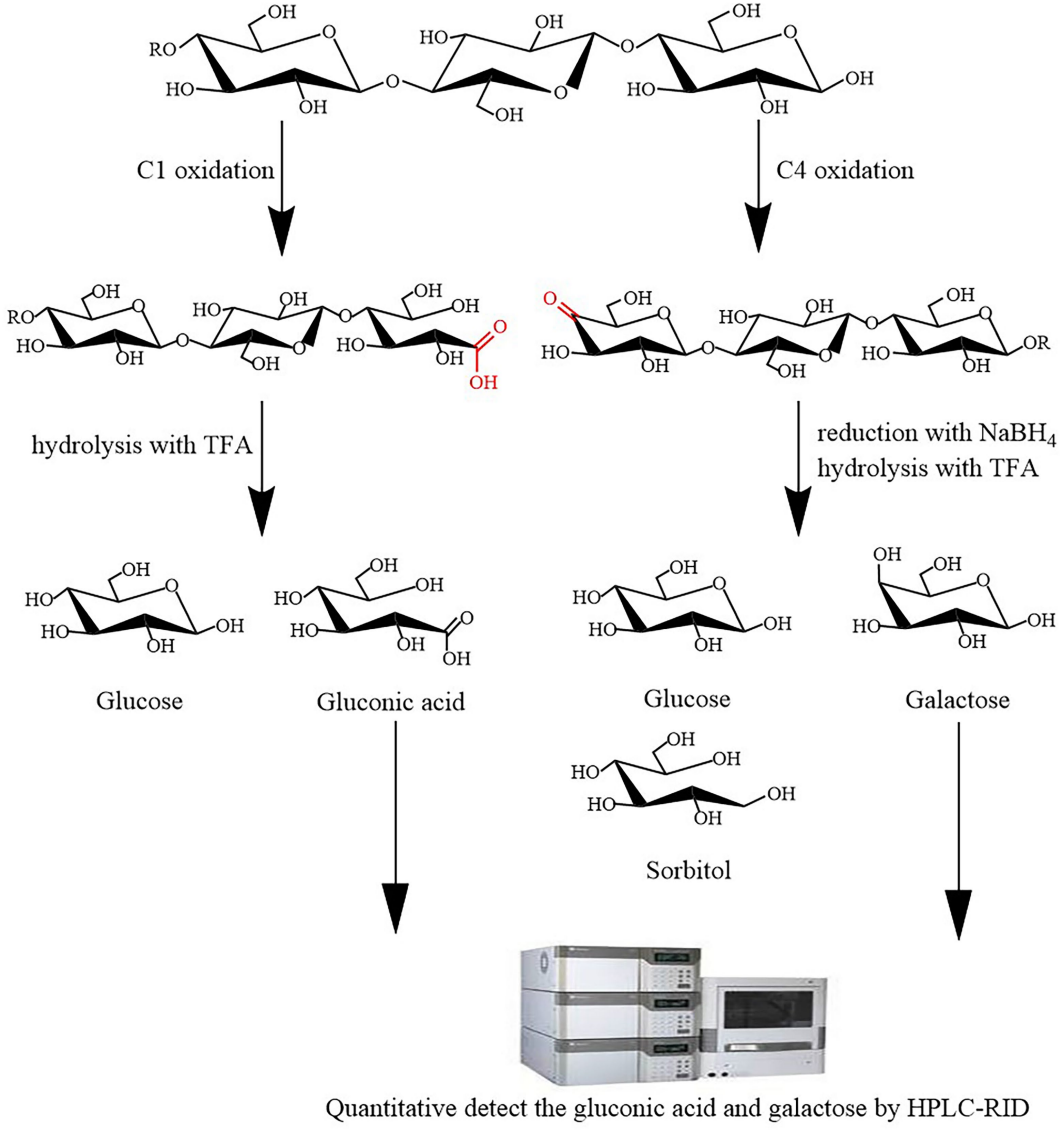
Schematic of the lytic polysaccharide monooxygenase (LPMO) activity assay. LPMO oxidatively cleaves cellulose to yield C1 and C4 oxidation products. Trifluoroacetic acid (TFA) can hydrolyze the products of LPMO C1 oxidation to yield glucose and gluconic acid, and the products of LPMO C4 oxidation can be reduced by NaBH4 and then hydrolyzed by TFA to yield glucose, galactose, and sorbitol. high-performance liquid chromatography–refractive index detector (HPLC-RID) can quantitative detect the resulting gluconic acid and galactose to estimate LPMO C1 and C4 oxidation activities.

The principle of LPMO activity assay is as follows: LPMOs employ O_2_ and/or H_2_O_2_ to oxidatively cleave crystalline cellulose, yielding C1 and C4 oxidation products. Therefore, LPMO activity can be determined using LPMO reaction products followed by hydrolysis with trifluoroacetic acid (TFA) and by reduction with NaBH_4_. TFA can hydrolyze the products of LPMO C1 oxidation to yield glucose and gluconic acid, and the products of LPMO C4 oxidation can be reduced by NaBH_4_ and then hydrolyzed by TFA to yield glucose, galactose, and sorbitol (Figure 1). HPLC-RID can detect the resulting gluconic acid and galactose to estimate LPMO C1 and C4 oxidation activities.

## Materials and methods

### Plasmids, strains, chemicals, and culture media

*Thermoascus aurantiacus* strain CGMCC3.17992 was isolated from horse manure in China according to our previous method (Li et al., 2003) and conserved in the China Microbial Strain Collection (Beijing, China). The plasmids pPICZαA and *P. pastoris* GS115 were purchased from Invitrogen. Avicel PH-101, ascorbic acid (Vc), glucose, gluconic acid, galactose, and sorbitol were purchased from Sigma. The culture medium mainly used low-salt LB medium, YPDS medium, BMGY, and BMMY medium.

### Analysis of the half-life of H_2_O_2_ at 50°C

The oxidative degradation of most thermophilic fungi AA9 LPMO takes place at 50°C, so the half-life of H_2_O_2_ at 50°C was determined with the addition of 10 mM ammonium acetate (pH 5.0) and 1 mM ascorbate to give a final concentration of 3% (v/v) H_2_O_2_. This experiment was carried out using the static method and the physical quantity chosen was volume. So that the H_2_O_2_ decomposition reaction takes place in a system with a fixed volume, the O_2_ given off during the reaction will increase the pressure in the system and the volume of gas during the reaction is read off through a measuring tube connected to the reaction bottle.

### Establishment of nTaAA9A activity assay

According to the principle of the LPMO activity assay, the reaction products of nTaAA9A were hydrolyzed by TFA and reduced by NaBH_4_ to yield gluconic acid and galactose. The activity of nTaAA9A was determined by quantification of gluconic acid and galactose using HPLC-RID. First, we quantified the gluconic acid standard and galactose standard at different concentrations (0.01, 0.02, 0.04, 0.08, 0.12, 0.16 mM) by HPLC-RID to make standard curves. Then, we collected the reaction products of nTaAA9A at different times points (6, 12, 24, 30, 36, 48 h) followed by hydrolysis with TFA and reduction with NaBH_4_ and quantified gluconic acid and galactose by HPLC-RID. Finally, the content of gluconic acid and galactose was calculated from the standard curve, and their production rates were used to estimate nTaAA9A C1 and C4 oxidation activities, respectively.

### cDNA cloning, expression vector construction, and *Pichia pastoris* transformation

Total RNA was isolated from *T. aurantiacus* mycelia using TRIzol reagent (Gibco), and RT-PCR amplified the cDNA of rTaAA9A with a pair of oligonucleotide primers following the RNA PCR Kit 3.0 instructions (Takara; Supplementary Table S1). A fragment of the rTaAA9A coding region without the signal peptide sequence was amplified by PCR using primers containing the *Xba*I and *Xho*I restriction sites. The primers were synthesized according to the gene (*ACS05720.1*) from the genomic sequencing of *T. aurantiacus* (Supplementary Table S1). The recombinant expression plasmid pPICZαA/*rTaAA9A* was obtained by digesting the pPICZαA plasmid with *Xho*I and *Xba*I and then ligating it with the amplification product. After linearization with *Pme*I, the recombinant expression plasmid was transformed into *P. pastoris* by electroporation. The transformants were evenly coated onto YPDS plates with 100 μg/ml of zeocin and were verified by DNA sequencing.

### Purification of rTaAA9A from *Pichia pastoris* and nTaAA9A from *Thermoascus aurantiacus*

We used the Pichia Expression System Kit Manual (Invitrogen) to induce and express the rTaAA9A protein in transformed *P. pastoris*. The transformed *P. pastoris* was cultured in BMMY medium containing 1 mM Cu^2+^ for 7 d at 28°C in shaking flasks. The protein was then purified by (NH_4_)_2_SO_4_ precipitation and His Trap column. (Chen et al., 2018). *Thermoascus aurantiacus* was cultured, fermented, and purified using the previous method (Yu et al., 2022) to obtain nTaAA9A. nTaAA9A was mainly purified using a DEAE-Sepharose columns and an Enrich SEC650 columns.

### Protein determination, SDS-PAGE, and carbohydrate staining

TaAA9A proteins were determined by the Lowry method (Lowry et al., 1951), and their purity was determined by sodium dodecyl sulfate polyacrylamide gel electrophoresis (SDS-PAGE; Laemmli, 1970). The carbohydrates in TaAA9A protein were stained according to Pierce® Glycoprotein Staining Kit (Thermo Scientific).

### TaAA9A activity assay

Phosphoric acid-swollen cellulose (PASC) was prepared according to the previous method (Phillips et al., 2011). Activity assay was performed by referring to the previous method (Chen et al., 2019). TaAA9A reaction products were formed following incubation of 0.5% PASC with 5 μM Cu^2+^-loaded TaAA9A in 10 mM ammonium acetate (pH 5.0) and 1 mM ascorbate at 50°C for 48 h. When PASC used as substrate, the reaction mixture was centrifuged at 8000 rpm at 4°C for 20 min. The supernatant was recovered and the soluble reaction products of rTaAA9A were analysed using thin-layer chromatography (TLC), Matrix-assisted laser desorption/ionization-time-of-flight tandem mass spectrometry (MALDI-TOF-MS), and HPLC-RID.

### TLC, MALDI-TOF-MS, and HPLC-RID

The reaction products of rTaAA9A and its mutated rTaAA9A were analyzed by TLC and MALDI-TOF-MS methods according to the previous methods (Vaaje-Kolstad et al., 2017; Chen et al., 2019). The reaction products and reduction products of TaAA9A were hydrolyzed by TFA (Horn et al., 2012) and then analyzed through the HPLC-RID method using an Agilent 1,200 high-performance liquid chromatography with a refractive index detector (RID). HPLC-RID analysis was performed on an Aminex HPX-87H ion exclusion column with a mobile phase of 5 mM H_2_SO_4_ at a flow rate of 0.3 ml/min, and the column temperature was 30°C. Glucose, gluconic acid, and sorbitol were analyzed according to the elution mode of standard glucose, gluconic acid, and sorbitol solutions.

### Permethylation of reaction products

Permethylation of reaction products was performed with improvement of the previous method (Needs and Selvendran, 1993). Using dimethyl sulfoxide as a solvent, the reaction product of dimethyl sulfoxide and sodium hydroxide was used as a strong base, and the methyl donor was methyl iodide. The entire operation was performed under nitrogen protection and anhydrous conditions, and the temperature was kept at 30°C. For hypermethylation, we added methyl iodide twice.

### Hydrolysis and reduction of reaction products

Hydrolysis and reduction of reaction products were performed with reference to the previous method (Beeson et al., 2012). TFA wad added to the reaction products to a concentration of 2.0 M, heated at 121°C for 1 h, dried under a stream of nitrogen and washed thrice with isopropanol. The reaction products were mixed with an equal volume of 20 mg/ml of NaBH_4_ solution dissolved in 1 M ammonia solution. Allow to react for 2 h at room temperature, shaking several times during this time. The reaction was terminated by the addition of glacial acetic acid (25–35 μl). The samples were then dried under a stream of nitrogen at 40°C. If blowing does not dry the sample, a methanol:acetic acid = 9:1 solution may be added, as appropriate. The resulting samples were hydrolysed by TFA.

### Site-directed mutagenesis

The sequence of rTaAA9A was analyzed using ClustalX2 and ESPript 3.0. According to the previous method, site-directed mutagenesis of rTaAA9A was performed (Chen et al., 2018). The primer sequences used for rTaAA9A site-directed mutagenesis are shown in the Supplementary Table S1.

## Results

### The half-life of H_2_O_2_ at 50°C

According to the above experimental principles and methods, the rate equation is derived as: ln(*V_∞_*-*V_t_*) = −0.0132 t-1.0433, *R*^2^ = 0.985 (Supplementary Figure S1).

So the reaction rate constant is 0.026 min^−1^, substituting *t*_1/2_ = 0.693/*k*, giving a half-life of 26.65 min. It can be seen that at a given temperature, the half-life is inversely proportional to the reaction rate constant, but not to the initial concentration of reactants. Furthermore, H_2_O_2_ is decomposed at 50°C, so using the previous method to assayed the activity of the thermophilic fungus AA9 LPMO would produce errors.

### Purification and activity assay of nTaAA9A

We used a native LPMO (nTaAA9A) from *T. aurantiacus* to establish an LPMO activity assay. Purification of nTaAA9A from culture filtrates of *T. aurantiacus* was grown in a cellulose-containing medium to homogeneity by ion-exchange chromatography and high-resolution gel filtration (Yu et al., 2022). SDS-PAGE was used to determine the molecular weight of purified nTaAA9A, at approximately 27 kDa (Supplementary Figure S2A), which was higher than the inferred amino acid sequence (24.39 kDa). In addition, periodic acid-Schiff staining confirmed the glycosylation of nTaAA9A (Supplementary Figure S2B), which is consistent with the previous results of nTaAA9A SDS-PAGE analyses (Yu et al., 2022).

**Figure 2:**
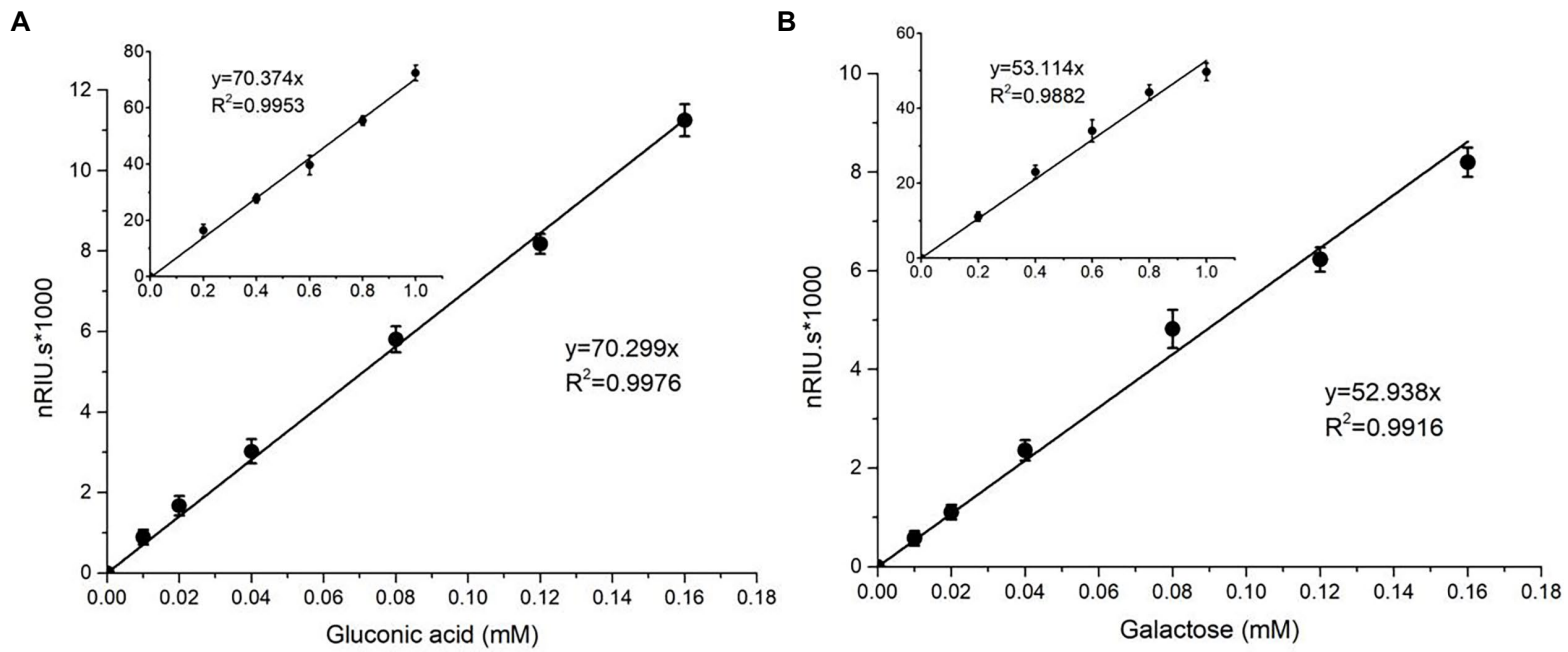
Standard curves of gluconic acid **(A)** and galactose **(B)** were analyzed using HPLC-RID. The standard curve ranges from 0.01 to 0.16 mM, and the inset is the same axial legend as the main graph, which shows the standard curve extending to 1.0 mM. All experiments were performed in three replicates, and error bars indicate the standard deviation.

**Figure 3:**
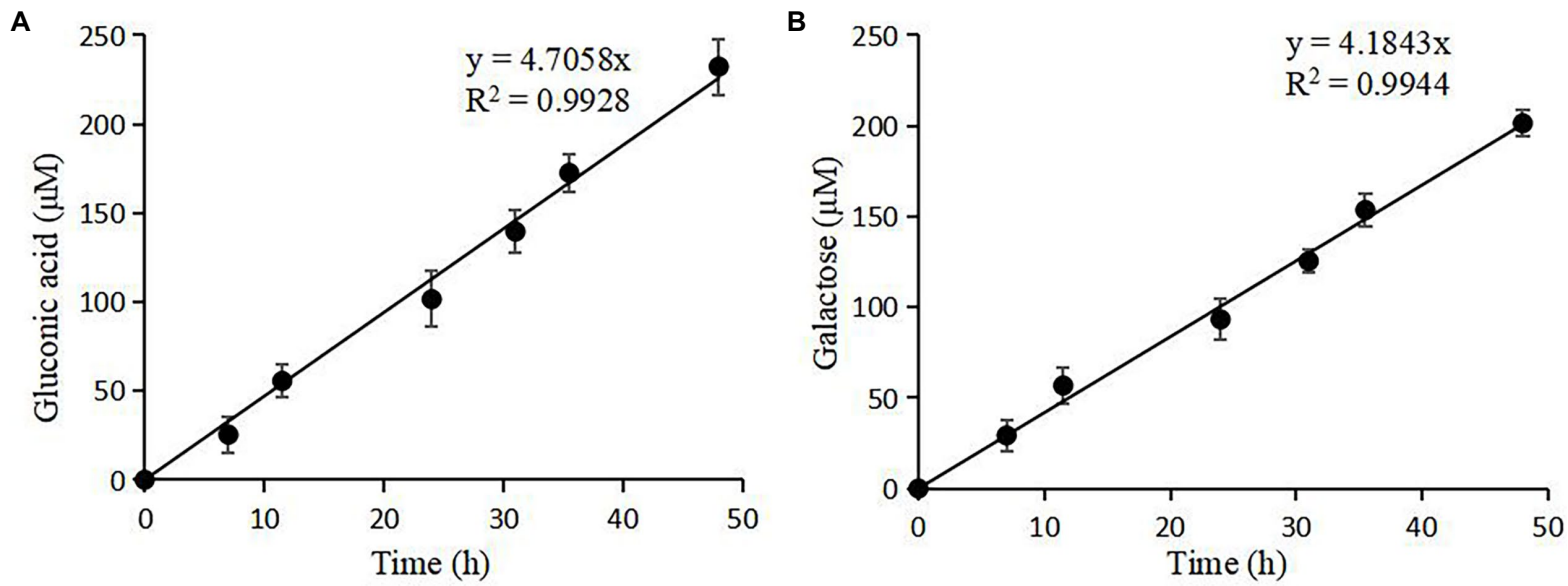
Quantification of the reaction products of nTaAA9A by HPLC-RID analysis. **(A)** TFA hydrolyzed the reaction products of nTaAA9A, and then gluconic acid was quantified with HPLC-RID analysis. **(B)** The reaction products of nTaAA9A were reduced by NaBH_4_ followed by hydrolysis with TFA. Then, galactose was quantified with HPLC-RID analysis. All experiments were performed in three replicates, and error bars indicate the standard deviation.

nTaAA9A soluble reaction products have C1-and C4-oxidized oligosaccharides (Wang et al., 2018), so the enzyme activity can be analyzed more comprehensively and accurately by both C1 and C4 oxidation. First, we quantified the gluconic acid standard and galactose standard at different concentrations by HPLC-RID to generate a standard curve (Figure 2). Then, we obtained nTaAA9A reaction products at different time points (6, 12, 24, 30, 36, 48 h) followed by hydrolysis with TFA and reduction with NaBH_4_. The activity of nTaAA9A was determined by quantifying gluconic acid and galactose by HPLC-RID. As shown in Figure 3, the formation of C1 and C4 oxidation products in nTAAA9A was linear over time. We defined the activity of LPMO based on the results; per unit of LPMO C1 and C4 oxidation activity was defined as the enzymatic amount of 1 μmol of gluconic acid or galactose released in 1 min. By this definition, the specific activities of nTaAA9A C1 and C4 oxidation were 0.646 and 0.574 U/mg, respectively.

### Identification of reaction products of rTaAA9A expressed in *Pichia pastoris*

To further analyze TaAA9A activity using this HPLC-RID method, we expressed TaAA9A in *P. pastoris* and identified TaAA9A’s reaction products. The *TaAA9A* gene was amplified from *T. aurantiacus* (Quinlan et al., 2011). To obtain TaAA9A protein, we expressed TaAA9A in *P. pastoris*. The expressed TaAA9A was named rTaAA9A. The Cu^2+^-loaded rTaAA9A expressed in *P. pastoris* was purified by a nickel column and subjected to SDS-PAGE electrophoresis (Supplementary Figure S3A).

We predicted one N-glycosylation site and five O-glycosylation sites in the amino acid sequence of TaAA9A using the NetNGlyc 1.0 server (services.healthtech.dtu.dk/service.php?NetNGlyc-1.0) and the NetOGlyc 4.0 server (services.healthtech.dtu.dk/service.php?NetOGlyc-4.0; Supplementary Table S2). The TaAA9A protein’s molecular mass was calculated to be 24.39 kDa. SDS-PAGE electrophoresis was used to estimate the molecular weight of rTaAA9A to be approximately 36 kDa. These observations suggest the presence of glycosylation through the recombinant TaAA9A protein expressed in *P. pastoris*. In addition, periodic acid-Schiff staining confirmed the glycosylation of rTaAA9A (Supplementary Figure S3B), consistent with the previous results of rTaAA9A SDS-PAGE analysis.

**Figure 4:**
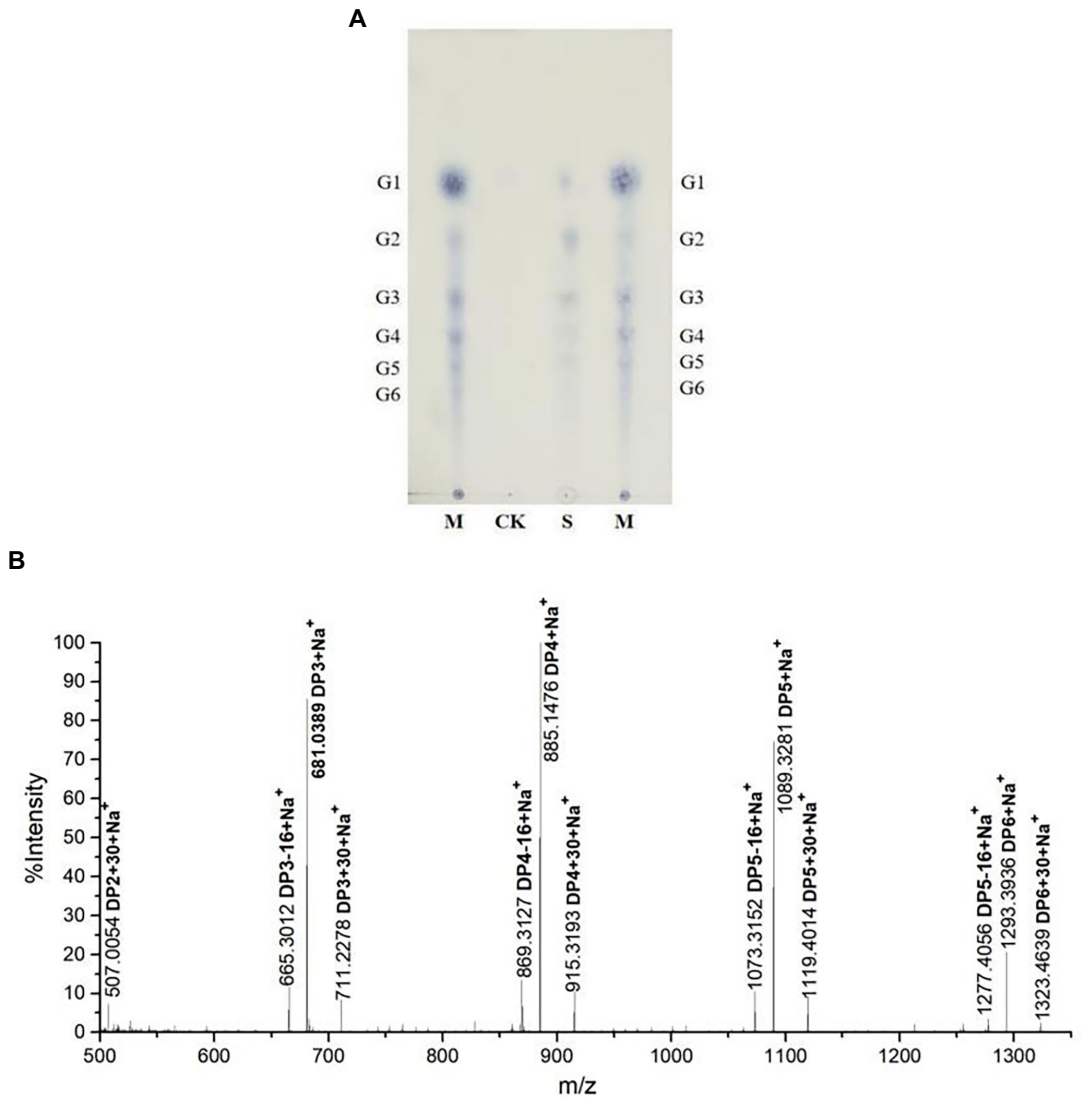
Analysis of rTaAA9A reaction products. **(A)** Analysis of rTaAA9A reaction products by TLC method. M, standard cellulosic oligosaccharides (G1–G6); S, rTaAA9A reaction products; CK, control sample was analyzed as above, except that rTaAA9A was not added. **(B)** Identification of the permethylation reaction products of rTaAA9A using MALDI-TOF-MS. C1-oxidized oligosaccharides (*m/z* + 30), C4-oxidized oligosaccharides (*m/z* − 16), and non-oxidized oligosaccharides (*m/z* + 0).

To identify rTaAA9A soluble products, we analyzed reaction products by TLC, MALDI-TOF-MS, and HPLC-RID methods. The TLC results indicated that rTaAA9A can cleave cellulose and yield cellulose oligosaccharides with various degrees of polymerization (DP; Figure 4A). Analysis of the permethylate reaction product of rTaAA9A using MALDI-TOF-MS showed that its m/z corresponds to the fibrillar oligosaccharides of DP3–DP6 (Figure 4B). Furthermore, the results showed that molecular ions at *m/z* + 30 and *m/z* − 16 corresponded to C1-and C4-oxidized oligosaccharides, indicating the oxidative properties of rTaAA9A.

**Figure 5:**
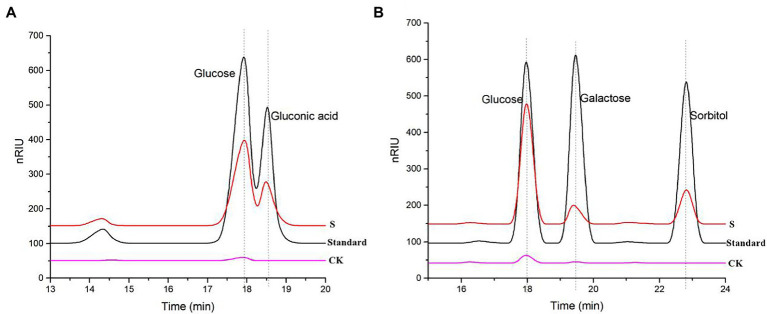
Analysis of rTaAA9A reaction products by HPLC-RID. **(A)** S, rTaAA9A reaction products hydrolyzed by TFA; CK, control sample, analyzed as described above, but without rTaAA9A. **(B)** S, rTaAA9A reaction products were reduced by NaBH_4_ followed by hydrolysis with TFA; CK, control sample was analyzed as above, except that rTaAA9A was not added. Gluconic acid and galactose were used as standards.

To further determine the C1 and C4 oxidation products, we adopted another chemical method, using rTaAA9A oxidation products hydrolyzed by TFA and reduced by NabH_4_ for HPLC-RID analysis. HPLC-RID results indicated the existence of C1-and C4-oxidized monosaccharides (Figure 5). This experiment further confirms that rTaAA9A oxidizes C1 and C4 on PASC substrates.

### Identification of reaction products of mutant rTaAA9A enzymes expressed in *Pichia pastoris*

To analyze the effect of aromatic residues on the substrate-binding plane on TaAA9A, we selected three aromatic residues to mutate on the substrate-binding plane by analyzing the sequence and structure of TaAA9A (Supplementary Figure S4), namely, Y24A, F43A, and Y212A. The methods of expression and purification were consistent with rTaAA9A, and SDS-PAGE electrophoresis was performed (Supplementary Figure S5), which was consistent with the size of rTaAA9A, indicating that mutant enzymes had been successfully expressed.

**Figure 6:**
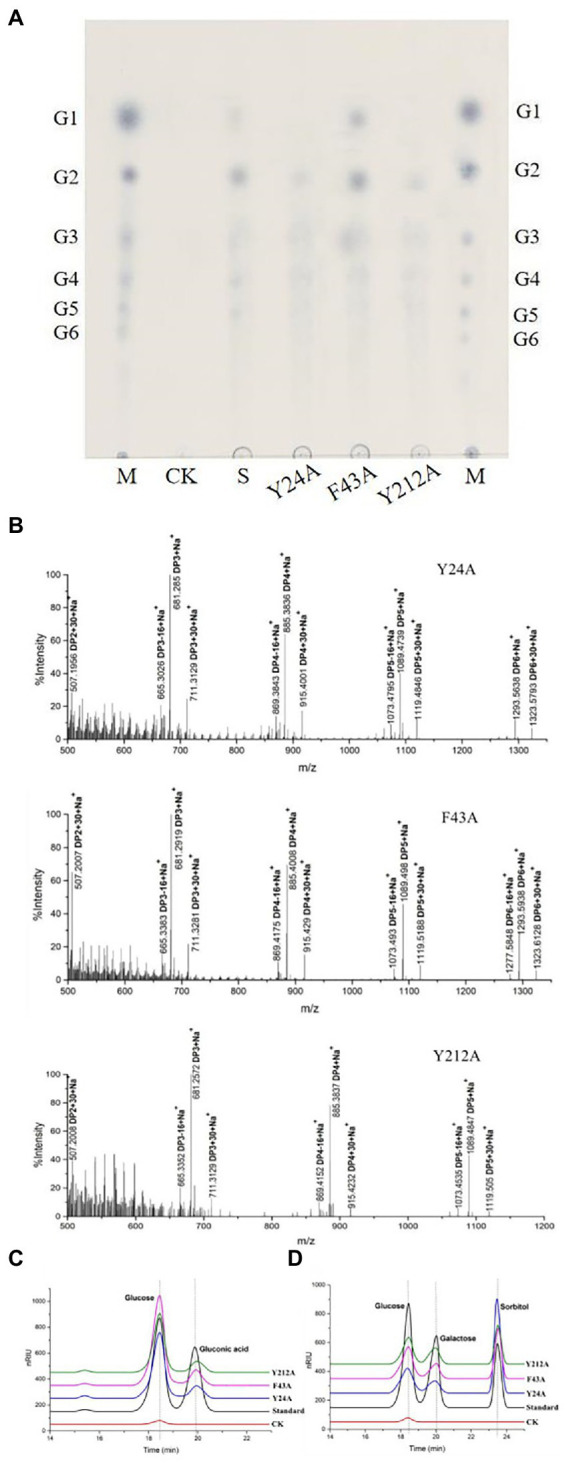
Identification of the mutated rTaAA9A reaction products using TLC and MALDI-TOF-MS and HPLC-RID. **(A)** M, standard cellulose oligosaccharides (G1–G6); S, rTaAA9A reaction products. **(B)** Identification of the permethylation reaction products of mutated rTaAA9A by MALDI-TOF-MS. The analysis method is the same as Figure 4. **(C)** Y24A, F43A, and Y212A reaction products were hydrolyzed by TFA. **(D)** Y24A, F43A, and Y212A reaction products were reduced by NaBH_4_, followed by hydrolysis with TFA. CK, control sample was analyzed as above, except that enzyme was not added. Gluconic acid and galactose were used as standards.

The reaction products of mutant rTaAA9A enzymes were identified using TLC analysis, MALDI-TOF-MS analysis, and HPLC-RID analysis as rTaAA9A. TLC showed that mutant rTaAA9A enzymes can cleave cellulose and yield cellulose oligosaccharides with various DP (Figure 6A). Analysis of the permethylate reaction product of mutant rTaAA9A enzymes using MALDI-TOF-MS showed that its m/z corresponds to the fibrillar oligosaccharides of DP3–DP5 (Figure 6B). The results also indicated that molecular ions at m/z + 30 and m/z − 16 corresponded to C1-and C4-oxidized oligosaccharides. HPLC-RID analysis indicated the existence of C1-oxidized monosaccharides (Figure 6C) and C4-oxidized monosaccharides (Figure 6D). These results indicated that mutant rTaAA9A enzymes oxidize C1 and C4 on PASC substrates.

### Activity assay of rTaAA9A and its mutated rTaAA9A

**Figure 7:**
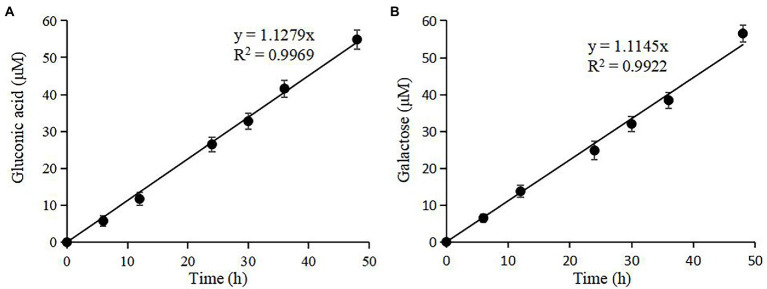
Quantification of rTaAA9A reaction products by HPLC-RID analysis. **(A)** TFA hydrolyzed the reaction products of rTaAA9A; then, gluconic acid was quantified with HPLC-RID analysis. **(B)** The reaction products of rTaAA9A were reduced by NaBH_4_ followed by hydrolysis with TFA, and then galactose was quantified using HPLC-RID. All experiments were performed in three replicates, and error bars indicate the standard deviation.

**Figure 8:**
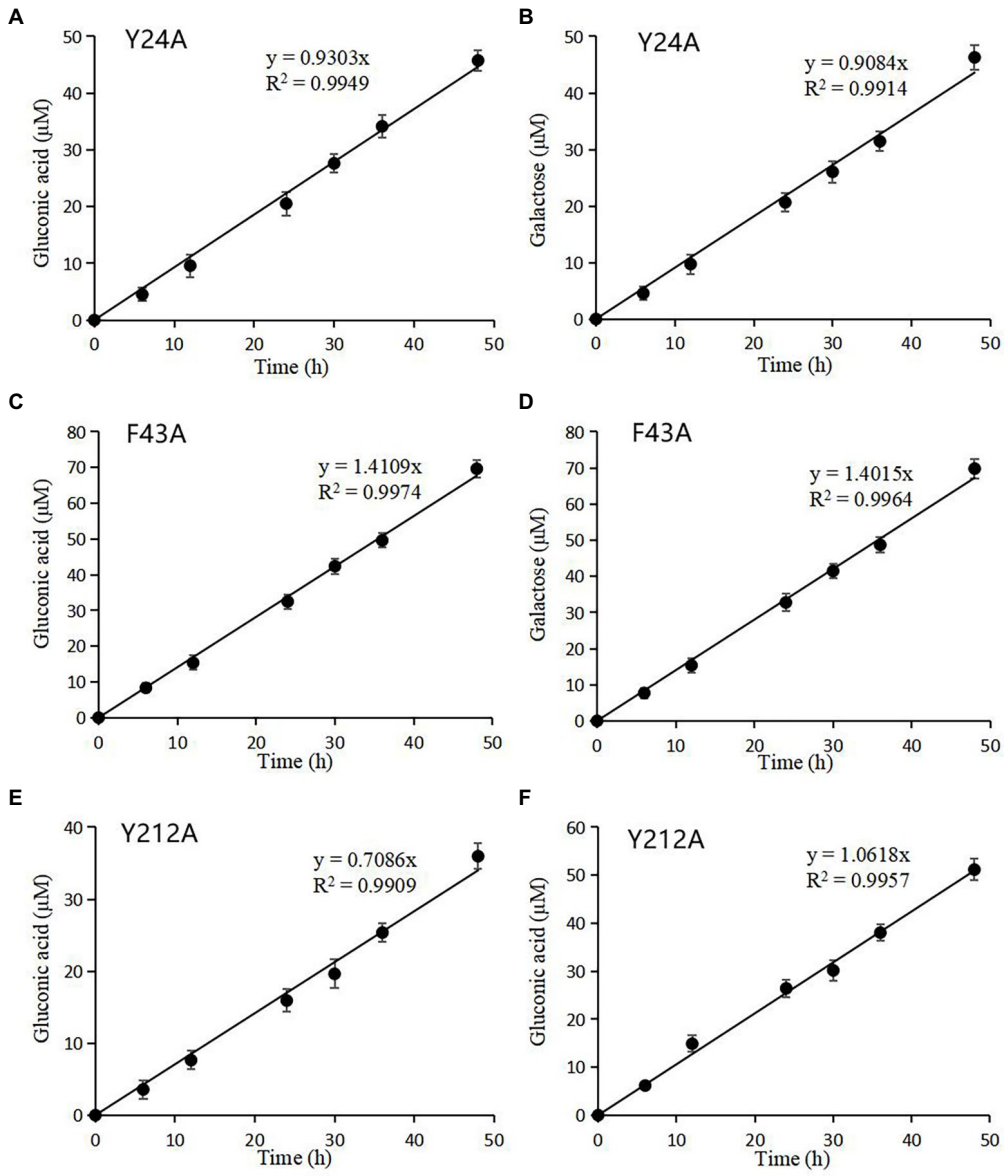
Quantification of mutated rTaAA9A reaction products by HPLC-RID analysis. Y24A **(A)**, F43A **(C)**, and Y212A **(E)** reaction products were hydrolyzed by TFA; then, gluconic acid was quantified using HPLC-RID. Y24A **(B)**, F43A **(D)**, and Y212A **(F)** reaction products were reduced by NaBH_4_ followed by hydrolysis with TFA and then quantified by HPLC-RID for galactose. All experiments were performed in three replicates, and error bars indicate the standard deviation.

C1-and C4-oxidized oligosaccharides were also found in rTaAA9A and its mutated rTaAA9A reaction products as nTaAA9A, so their enzyme activities could be analyzed and the validity of the established activity assay could be verified. As shown in Figure 7, the formation of both C1 and C4 oxidation products of rTaAA9A had a linear relationship over time. The specific activities of rTaAA9A C1 and C4 oxidation were 0.155 and 0.153 U/mg, respectively. As shown in Figure 8, the activity of F43A increased compared to rTaAA9A, and the specific activities of F43A C1 and C4 oxidation were 0.194 and 0.192 U/mg, respectively. The specific activities of Y24A C1 and C4 oxidation were 0.128 and 0.125 U/mg, respectively. The specific activities of Y212A C1 and C4 oxidation were 0.097 and 0.146 U/mg, respectively.

## Discussion

An accurate and comprehensive method for the determination of LPMO activity can screen out highly active LPMOs, as well as better study their properties and catalytic mechanisms (Kittl et al., 2012; Wu et al., 2022). The previous methods for activity assay mainly focus on the side activity of LPMO, mostly by quantifying the formation of H_2_O_2_ to assay the activity of LPMO (Kittl et al., 2012; Wang et al., 2018; Wu et al., 2022), but H_2_O_2_ is unstable under high-temperature conditions. Furthermore, it is difficult to pool all the different oligosaccharides produced by LPMO for assaying by the existing methods (Breslmayr et al., 2018, 2019; Brander et al., 2021; Wang et al., 2021). Therefore, it is difficult for existing methods to accurately assay the LPMO activity of thermophilic fungi. The activity assay method established in this study can directly target AA9 LPMO of thermophilic fungi under high-temperature conditions, and it can assay the activity of C1, C4, and C1/C4 type LPMOs, making it more accurate and comprehensive. Only two reagents, TFA and NaBH_4_, are used in the assay, which do not affect LPMO activity, and the method is not affected by other cellulases. Although this activity detection method is time-consuming, it can compare the differences in C1 and C4 oxidation of LPMOs from different origins, which makes it a useful tool to compare the activities of different types of LPMOs and can be used to detect the enzymatic properties of LPMOs, which can provide a theoretical basis for exploring the oxidation mechanism of LPMO.

We consider that the AA9 LPMO activity assay established in this study has the following advantages: (1) the method is simple, accurate, and comprehensive because different LPMO oligosaccharides are accumulated to glucose, gluconic acid and galactose; (2) the method directly assays the primary activity of AA9 LPMO from thermophilic fungi; (3) the method can assay the activities of C1-type, C4-type, and C1/C4-type LPMOs; (4) no substances affecting the activity are added; (5) no fear of contamination by other cellulases; and (6) no expensive instrumentation is required except for HPLC-RID.

In fungal AA9 LPMOs, the Nε position of the natural N-terminal first amino acid histidine is modified by methylation, but this modification is not present in fungal LPMOs expressed in *P. pastoris* (Petrović et al., 2018). Our study shows that the C1 and C4 oxidation of rTaAA9A decreased 4.168 and 3.752 times lower than that of nTaAA9A, respectively. The absence of such modification in rTaAA9A expressed in *P. pastoris* may be one reason for the significant difference in the specific activity of nTaAA9A and rTaAA9A. It was reported that the methylation of the N-terminal first amino acid histidine in TaAA9A could protect itself from reactive oxygen species generated in the active site (Petrović et al., 2018). In addition, we found many kinetic studies on the oxidation of cellulose by LPMO with H_2_O_2_ as co-substrate (Kont et al., 2020; Frandsen et al., 2021; Kuusk and Väljamäe, 2021). Comparing the activity of TaAA9A with that of other LPMO, we found that fungus-derived LPMO showed high catalytic efficiency, and H_2_O_2_-driven LPmos had important biological significance for cellulose degradation. However, LPMO of thermophilic fungi degrades cellulose at a temperature above 50°C, at which H_2_O_2_ is unstable, and errors will occur in the analysis of LPMO activity. This effect can be excluded by our method.

There is evidence that aromatic residues on the substrate-binding plane of LPMOs play a role in substrate-binding and determine the regioselectivity of LPMOs (Bennati-Granier et al., 2015; Borisova et al., 2015; Frandsen et al., 2016; Moses et al., 2016; Simmons et al., 2017). Our current study suggest that a single site mutation has little effect on the regioselectivity and little change in activity, probably because TaAA9A has a stable binding state and the structure remains stable after a single site mutation (Vu et al., 2014; Danneels et al., 2019). Interestingly, Y212A mutation alters the regioselectivity of rTaAA9A and loses most of its C1 oxidation activity. It is possible that mutation in Tyr212 alters the binding surface close to the site +2/+3 (Supplementary Figure S6), resulting in a regioselective change in rTaAA9A (Courtade et al., 2016; Yu et al., 2022).

## Conclusion

In the current study, a method based on HPLC-RID has been successfully established for assaying the TaAA9A activity of the thermophilic fungus *T. aurantiacus*. The method provides an effective activity assay method for LPMOs of thermophilic fungi and can compare the differences in C1 and C4 oxidation. This demonstrates the importance of this activity assay for studying AA9 LPMOs from thermophilic fungi.

## Data availability statement

The original contributions presented in the study are included in the article/Supplementary material, further inquiries can be directed to the corresponding author.

## Author contributions

WY and DL designed the experiments, analyzed the data, and contributed to the drafting and revision of the manuscript. WY, JY, and DL performed the experiments. All authors contributed to the article and approved the submitted version.

## Funding

This research was funded by the National Natural Science Foundation of China (Grant No. 32271893) and the Ministry of Science and Technology of China (Grant No. 2015BAD15B05).

## Conflict of interest

The authors declare that the research was conducted in the absence of any commercial or financial relationships that could be construed as a potential conflict of interest.

## Publisher’s note

All claims expressed in this article are solely those of the authors and do not necessarily represent those of their affiliated organizations, or those of the publisher, the editors and the reviewers. Any product that may be evaluated in this article, or claim that may be made by its manufacturer, is not guaranteed or endorsed by the publisher.

## Supplementary material

The Supplementary material for this article can be found online at: https://www.frontiersin.org/articles/10.3389/fmicb.2022.1063025/full#supplementary-material

## Footnotes

^1^ www.cazy.org

## Abbreviations

AA9: Auxiliary activities family 9
LPMOs: Lytic polysaccharide monooxygenases
SDS-PAGE: Sodium dodecyl sulfate polyacrylamide gel electrophoresis
TLC: Thin-layer chromatography
DP: Degree of polymerization
MALDI-TOF-MS: Matrix-assisted laser desorption/ionization-time-of-flight tandem mass spectrometry
HPLC-RID: High-performance liquid chromatography–refractive index detector
PASC: Phosphoric acid-swollen cellulose
TFA: Trifluoroacetic acid
