# Supplementary material for: Analysis of lytic polysaccharide monooxygenase activity in thermophilic fungi by high-performance liquid chromatography–refractive index detector
Source: Front Microbiol. 2022 Nov 22;13:1063025. doi: 10.3389/fmicb.2022.1063025 (PMC9722967; doi:10.3389/fmicb.2022.1063025)
Supplement: Supplementary file 1 [file Data_Sheet_1.docx]

**Analysis of lytic polysaccharide monooxygenase activity in thermophilic fungi by high-performance liquid chromatography-refractive index detector**


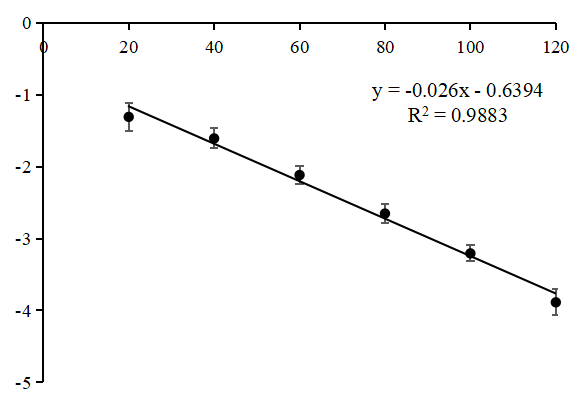


**Figure S1** Rate constant for the decomposition of H_2_O_2_ at 50 ℃.

**
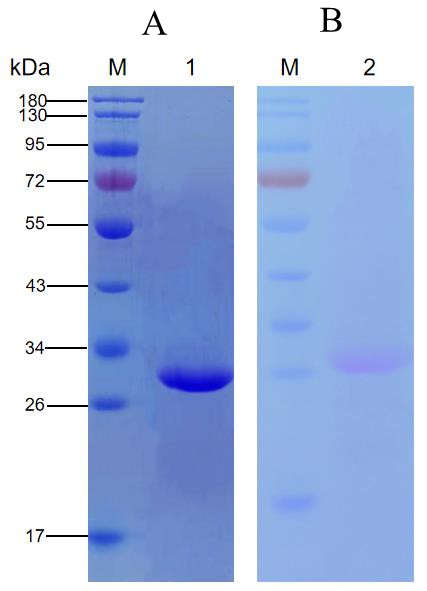
**

**Figure S2** SDS-PAGE of *T. aurantiacus* nTaAA9A. nTaAA9A was observed with Coomassie brilliant blue (A) and Pierce glycoprotein staining kit (B). Lane M, marker (kDa); Lanes 1 and 2, nTaAA9A.


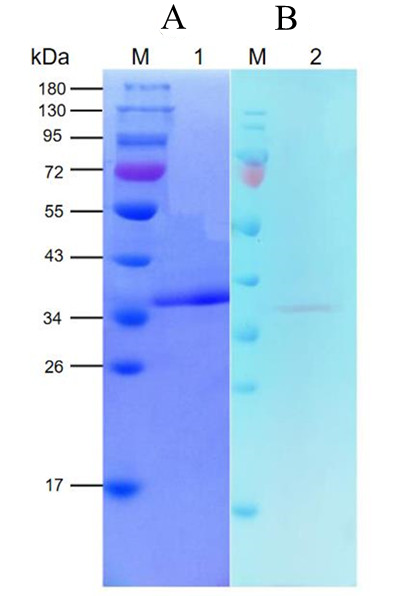


**Figure S3** SDS-PAGE of rTaAA9A. rTaAA9A was observed with Coomassie brilliant blue (A) and Pierce glycoprotein staining kit (B). Lane M, marker (kDa); Lanes 1 and 2, rTaAA9A.


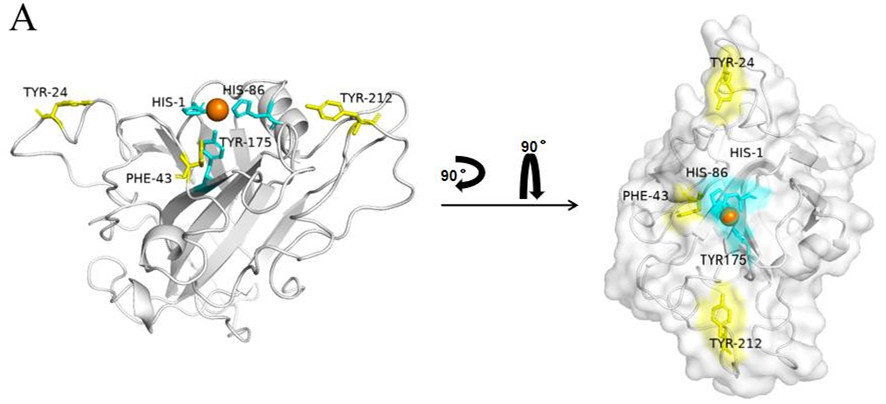

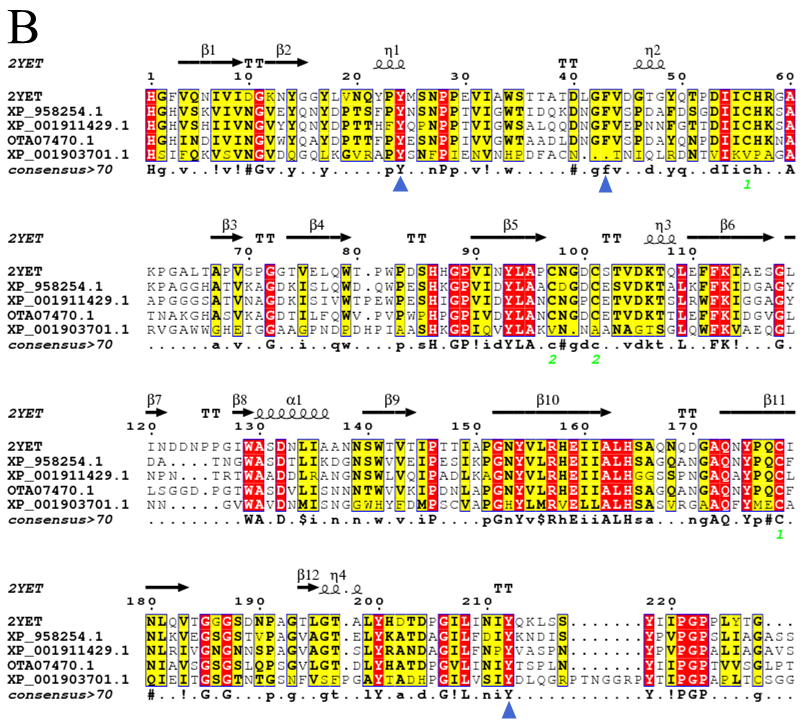


**Figure S4** Analysis of rTaAA9A sequence and structure. (A) Analysis of rTaAA9A structure using PyMOL. The conserved residues adjacent to the copper are colored in cyan. The aromatic residues are colored in yellow. The copper ion is shown as an orange sphere; (B) Sequence analysis using ClustalX2 and ESPript 3.0, including *Neurospora crassa* (XP_958254.1), *Podospora anserina* (XP_001911429.1), *Trichoderma parareesei* (OTA07470.1), *Podospora anserina* (XP_001903701.1). Mutated amino acid residues are marked with solid triangles in blue.


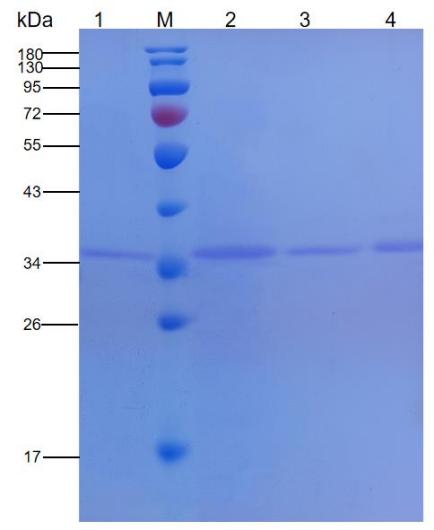


**Figure S5** SDS-PAGE of rTaAA9A and mutated rTaAA9A. Lane M, marker (kDa); lane 1, rTaAA9A; lane 2, Y24A; lane 3, F43A; lane 4, Y212A.


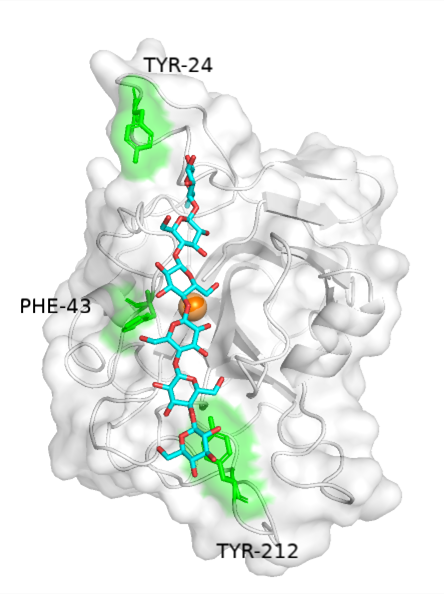


**Figure S6** Homology model of rTaAA9A binding with cellohexaose. rTaAA9A model was aligned to CvAA9_A (PDB ID: 6yde) using PyMOL. The aromatic residues are colored in green. The copper ion is shown as an orange sphere. The carbon atoms of cellohexaose are colored in cyan, and oxygen atoms of cellohexaose are colored in red.

**Table S1** List of primers used for PCR of the rTaAA9A protein.

| Primer | Sequence (5’——3’) | Purpose |
| --- | --- | --- |
| rTaAA9A-cF | ATGTCCTTTTCCAAGATA | ORF cDNA of rTaAA9A |
| rTaAA9A-cR | ACCAGTATACAGAGGAGG | ORF cDNA of rTaAA9A |
| rTaAA9A-F | AGGGGTATCTCTCGAGAAAAGACATGGCTTCGTTCAG | Expression of rTaAA9A |
| rTaAA9A-R | GAGTTTTTGTTCTAGAGCACCAGTATACAGAGGAGGA | Expression of rTaAA9A |
| Y24A-F | GTGAACCAGTATCCAGCCATGTCCAATCCTCCA | Y24A mutagenesis |
| Y24A-R | GGCTGGATACTGGTTCACTAGATACCCGCCATA | Y24A mutagenesis |
| F43A-F | GCAACTGATCTTGGAGCCGTGGACGGTACTGGA | F43A mutagenesis |
| F43A-R | GGCTCCAAGATCAGTTGCCGTAGTAGACCAGGC | F43A mutagenesis |
| Y212A-F | ATTCTGATCAACATCGCCCAGAAACTTTCCAGC | Y212A mutagenesis |
| Y212A-R | GGCGATGTTGATCAGAATTCCAGGATCGGTATG | Y212A mutagenesis |

**Table S2** The putative potential N-linked and O-linked glycosylation sites of rTaAA9A. A list of potential glycosylation sites showed their positions in the sequence and the prediction confidence scores. Only the sites with scores higher than 0.5 are predicted to be glycosylated.

|  | position | scores |
| --- | --- | --- |
| N-glycosylation sites | 138 | 0.5667 |
| O-glycosylation site | 35 | 0.5761 |
|  | 37 | 0.5275 |
|  | 39 | 0.6011 |
|  | 47 | 0.5155 |
|  | 144 | 0.5262 |
